# The AlphaFold Database of Protein Structures: A Biologist’s Guide

**DOI:** 10.1016/j.jmb.2021.167336

**Published:** 2022-01-30

**Authors:** Alessia David, Suhail Islam, Evgeny Tankhilevich, Michael J.E. Sternberg

**Affiliations:** Centre for Integrative System Biology and Bioinformatics, Department of Life Sciences, Imperial College London, London SW7 2AZ, UK

**Keywords:** AlphaFold, human proteome, three-dimensional model, inter-domain accuracy

## Abstract

AlphaFold, the deep learning algorithm developed by DeepMind, recently released the three-dimensional models of the whole human proteome to the scientific community. Here we discuss the advantages, limitations and the still unsolved challenges of the AlphaFold models from the perspective of a biologist, who may not be an expert in structural biology.

## Highlights

• AlphaFold, released the three-dimensional models (3D) of the human proteome.
• Accurately and poorly modelled regions may be present within a single AlphaFold model.
• The advantages, limitations and unsolved challenges of AlphaFold models are discussed.

## Graphical abstract

**Figure:**
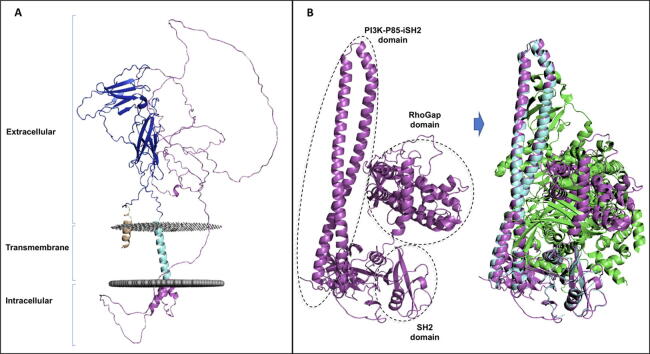

In July 2021, the predicted three-dimensional models for the whole human proteome generated using AlphaFold, the deep learning algorithm developed by DeepMind, were made available to the public, as recently reported in Nature.^1^ In the absence of an experimental structure, computational methods have been used for decades to predict three-dimensional protein models. Before the advent of the AlphaFold algorithm, the main approaches were homology modelling and *ab-initio*. In homology modelling (or template-based approach), which was the most successful and widely used approach, a model is built based on the experimental structure of a homologue, which serves as a structural template. In the *ab-initio* method (or template-free approach), the model is built by using physics-based and/or knowledge-based energy functions, combined with evolutionary information, which are used to generate distance (or contact) maps.^2^

The deep neural network of the AlphaFold algorithm, which combines features derived from homologous templates and from multiple sequence alignment to generate the predicted structure, has shown an outstanding accuracy in predicting the three-dimensional structure of proteins with otherwise unknown fold. In CASP14, which is a blind trial that critically assesses techniques for protein structure prediction, AlphaFold (which entered the blind trial under the name AlphaFold2, to distinguish this from an earlier version), markedly outperformed other protein structure modelling methods. When using the root mean square deviation (rmsd), a commonly used method to measure the similarity between two structures (the lower the score the more similar the structures), AlphaFold models had a median backbone accuracy of 0.96 Å rmsd compared to 2.80 Å rmsd of the next best performing method. AlphaFold models also had a high level of accuracy in predicting the position of residue side chains when the protein backbone prediction was accurate.^3^ The leading edge performance of AlphaFold is confirmed by the on-going Continuous Automated Model EvaluatiOn (CAMEO).^4^ In light of this remarkable achievement, DeepMind made the entire set of models for the human proteome freely available to the scientific community, available at https://alphafold.ebi.ac.uk/ and hosted by the European Bioinformatics Institute.

From the perspective of the biologist and the non-expert in the structural biology field, what are the advantages, the limitations and the still unsolved challenges of the models generated by AlphaFold? Currently <10% of the proteins in the human proteome have at least some experimentally-obtained coordinates (protein-level coverage) and ∼17% of the residues in the human proteome can be mapped to an experimental structure (residue-level coverage) (4). In the AlphaFold database, the protein-level coverage for the human proteome is 98.5%. However, only 58% of residues are modelled with high confidence, defined as a predicted local distance difference test score [pLDDT] > 70.^1^ This 58% high confidence residue-level coverage is an overall improvement of <10% compared to the combined coverage of experimental structures and models generated using templates with sequence identity >30% and standard template modelling predictors (∼50% residue-level coverage).,^5^, ^6^ However, this increment of coverage will be transformative by providing models which would not be otherwise available to the community. Moreover, the improved accuracy of AlphaFold models compared to template-based ones will be important in several applications, including structure-based drug discovery,^7^ variant prediction and to assist experimental structure determination (e.g. molecular^8^)^9^, ^10^, ^11^, ^12^ (and extensively discussed in the JMB AlphaFold Special Issue, Volume 433, Issue 20, 1st October 2021). However, in cases where the predicted model of the holo form with its cognate ligand is important, a less accurate model which inherits the ligand coordinates from the template may provide more biological insights compared to a more accurate AlphaFold model of the apo form. At present, the models released by AlphaFold do not allow user selection of the appropriate ligand-bound template, which is facilitated by many of the traditional template-based methods.^13^, ^14^

This relatively small improvement in coverage is not surprising given that 37–50% of the human proteome is predicted to be structurally disordered.^15^ Disordered protein domains are often important for intracellular signalling and can transition from a disordered to an ordered state, e.g. upon binding to other proteins. Predicting how these amino acid sequences fold remains a challenge.^16^ In the AlphaFold models, these disordered regions are identified by a pLDDT < 50 and are often graphically presented as long filaments.

Another major challenge in the field of structural biology and protein modelling is the identification of the correct placement of domains in a multi-domain protein, also known as inter-domain accuracy. AlphaFold provides full chain models for >98% of human proteins, many of which are multidomain. In CASP14, the AlphaFold inter-domain accuracy was good (formally 70% of models having a template modelling (TM) score > 0.7). Domains are often connected by short and flexible stretches of amino acids, known as linkers, which allow domains to undergo conformational changes in response to biological stimuli. In the AlphaFold models, these linkers are not always predicted at high confidence (pLDDT > 70). The implication of this is that the spatial placement, and in some cases, proximity of two ordered domains should be interpreted with caution. Here, we wish to highlight the need to inspect the heat map or “predicted aligned error” provided by AlphaFold that displays the model’s inter-domain accuracy, which should always be considered alongside the per-residue pLDDT score when interpreting model accuracy. Additionally, the relative position of domains should be explored using biological data, For example, by using experimental structures with lower resolution, structures of homogous proteins or of complexes with partial coverage of the protein sequence.

We illustrate the challenge of positioning domains with two examples. Figure 1(A) shows the predicted structure for the growth hormone receptor, where the long disordered intracellular tail is placed next to the ordered extracellular domain. Figure 1(B) shows that the relative location of the domains in PIK3R1 is inconsistent with the experimental structure of the PIK3R1 / PIK3CD complex with major clashes between chains. In this example, the relative positions of the PIK3R1 domains may alter between the single chain and the complex. Hence, if the links between the domain are flexible, AlphaFold could be generating a correct model for the single chain or be generating one of an ensemble of domain conformations.

**Figure 1:**
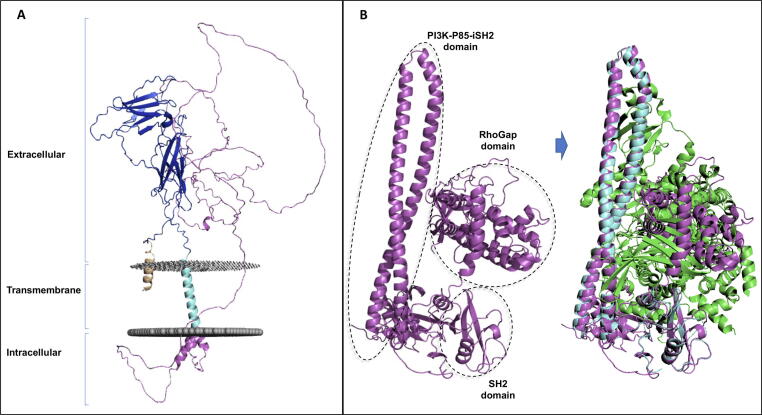
**The challenges of protein structure prediction**. A) AlphaFold model of the growth hormone receptor (GHR, UniProt P10912). The long, unstructured intracellular tail of the growth hormone receptor (residues 289–638) is presented in magenta as a long filament and is wrongly placed next to the extracellular domain. The extracellular domain (residues 19–264) is presented in blue and the transmembrane domain (residues 265–288) in cyan. B) On the left, AlphaFold model of the PIK3R1 protein (in magenta, UniProt P27986). The main domains of PIK3R1 are highlighted with dotted lines. On the right, the AlphaFold model of PIK3R1 (in magenta) is superposed to the experimental structure of PIK3R1 (in cyan) in complex with PIK3CD (in green; PDB 5M6U). The PIK3R1 interdomain placement would results in a steric clash with PIK3CD. PI3K-P85-iSH2, Phosphatidylinositol 3-kinase regulatory subunit P85 inter-SH2 domain.

Another challenge for protein structure predictions is that several proteins are very long. Currently the AlphaFold database on the EBI website does not include models for proteins longer than 2700 residues. Thus, no models are available for 207 large (residue range 2701–34350), biologically important human proteins, such as those encoded by *Titin* and *Dystrophin*, the main genes responsible for congenital cardiomyopathy^17^ and muscular dystrophy.^18^ However, AlphaFold has generated several overlapping model fragments for these proteins (available for download at https://alphafold.ebi.ac.uk/download). Inevitably, interpreting models for very long proteins will be difficult.

The structural coverage of the human proteome is not uniform. A recent study showed that some classes of proteins, such as drug targets, have been studied better than others and their structural coverage at protein level is already very high.^19^ We explored the additional value of AlphaFold models compared to the coverage that can be obtained using standard homology modelling algorithms, such as Phyre2,^13^ on two sets of proteins that make a fundamental contribution to morbidity and mortality: the top 25 cancer proteins from the PanCan TumorPortal database^20^ and the top 5 proteins causing familial hypercholesterolemia, one of the main inherited causes of premature cardiovascular disease (https://panelapp.genomicsengland.co.uk/panels/772/).^21^ Of these 30 proteins, 8 are longer than 2700 residues and models are not provided for these on the EBI website. For the remaining 22 proteins, the additional coverage at residue level provided by AlphaFold models (pLDDT > 70) over standard homology methods, exemplified by Phyre2, was not substantial: 13,059 versus 13,214 (Table 1).

**Table 1:** **AlphaFold database coverage compared to the experimental coverage and the coverage obtained using standard homology-based methods exemplified by our *in house* program Phyre2.** The three-dimensional coordinate files were extracted from the ProteinDataBank (PDB). Phyre2 was used as a representative of homology-based methods. Only Phyre2 models with a confidence score >98% and sequence identity >30% were selected. For AlphaFold models, the residue coverage is presented according to the per-residue pLDDT score.

			Experimental coverage		AlphaFold (pLDDT ≥ 70)		Phyre2 (Confidence > 98%; Seq ID > 30%)	
Gene	UniProt Id	Protein length	residues, n.	residues, %	residues, n.	residues, %	residues, n.	residues, %
*LDLRAP1*	Q5SW96	308	16	5.2	159	51.6	144	46.8
*SETD2*	Q9BYW2	2564	424	16.5	513	20.0	345	13.5
*CREBBP*	Q92793	2442	556	22.8	823	33.7	829	33.9
*ARID1A*	O14497	2285	586	25.6	554	24.2	647	28.3
*NOTCH1*	P46531	2555	797	31.2	602	23.6	551	21.6
*SMARCA4*	P51532	1647	682	41.4	831	50.5	945	57.4
*PBRM1*	Q86U86	1689	879	52.0	1126	66.7	373	22.1
*BRAF*	P15056	766	447	58.4	421	55.0	295	38.5
*FBXW7*	Q969H0	707	444	62.8	471	66.6	443	62.7
*VHL*	P40337	213	160	75.1	155	72.8	150	70.4
*RB1*	P06400	928	698	75.2	592	63.8	763	82.2
*LDLR*	P01130	860	705	82.0	643	74.8	650	75.6
*PTEN*	P60484	403	334	82.9	315	78.2	353	87.6
*EGFR*	P00533	1210	1010	83.5	860	71.1	914	75.5
*TP53*	P04637	393	340	86.5	227	57.8	357	90.8
*KRAS*	P01116	189	171	90.5	175	92.6	189	100.0
*PCSK9*	Q8NBP7	692	642	92.8	563	81.4	622	89.9
*MTOR*	P42345	2549	2370	93.0	2074	81.4	2533	99.4
*APOE*	P02649	317	298	94.0	218	68.8	299	94.3
*PIK3R1*	P27986	724	683	94.3	621	85.8	596	82.3
*PIK3CA*	P42336	1068	1061	99.3	1002	93.8	1060	99.3
*CDKN2A*	P42771	156	156	100.0	114	73.1	156	100.0
**TOTAL**					13,059		13,214	
*NF1*	P21359	2839			NA	NA	595	21.0
*APC*	P25054	2843			NA	NA	571	20.1
*ATM*	Q13315	3056			NA	NA	3053	99.9
*SPEN*	Q96T58	3664			NA	NA	456	12.4
*APOB*	P04114	4563			NA	NA	0	0.0
*FAT1*	Q14517	4588			NA	NA	518	11.3
*MLL3*	Q8NEZ4	4911			NA	NA	156	3.2
*MLL2*	O14686	5537			NA	NA	309	5.6

NA, AlphaFold model not available from the EBI website. However, the predicted overlapping segments for these long proteins can be downloaded from https://alphafold.ebi.ac.uk/download.

*LDLR, APOB, APOE, PCSK9* and *LDLRAP1* cause Familial Hypercholesterolemia. The remaining 25 genes are the top 25 genes from PanCan (4742 patients) in the TumorPortal.

Seq ID, sequence identity between query and template.

In conclusion, the AlphaFold algorithm has rightly been called a “game changer” in the field of structural biology and has demonstrated one of the many applications of deep learning algorithms in biomedicine.^22^, ^23^ However, AlphaFold has not completely solved the “protein folding problem” and many challenges remain, such as predicting the relative position of domains within a chain, how domains shift their relative conformation in response to stimuli, and how domains transition from disorder to order.

## Author contribution

AD and SI performed model analysis. AD wrote the first draft of the manuscript. All authors contributed to the interpretation of findings and manuscript preparation. All authors approved the final version of the manuscript.

## Disclosures

AD is supported by Wellcome Trust (grant 218242/Z/19/Z).

ET is supported by a BBSRC grant to Imperial College London (BB/M011178/1).

These Funders and DeepMind had no role in the conceptualization, design, data collection, analysis, decision to publish or preparation of the manuscript.

This research was funded in whole, or in part, by the Wellcome Trust grant number 218242/Z/19/Z. For the purpose of open access, the author has applied a CC BY public copyright licence to any Author Accepted Manuscript version arising from this submission.

## Conflict of interest

DeepMind are proving funding for Master studentships at Imperial College London including potentially for a course of which MJES is the Director. The Authors declare no other competing interests.
